# Activation of the Sphingosine 1 Phosphate–Rho Pathway in Pterygium and in Ultraviolet-Irradiated Normal Conjunctiva

**DOI:** 10.3390/ijms20194670

**Published:** 2019-09-20

**Authors:** Nozomi Igarashi, Megumi Honjo, Takashi Fujishiro, Tetsuya Toyono, Takashi Ono, Yosai Mori, Kazunori Miyata, Hideru Obinata, Makoto Aihara

**Affiliations:** 1 Department of Ophthalmology, Graduate School of Medicine, The University of Tokyo, Tokyo 113-8655, Japan; kindenauthor@yahoo.co.jp (N.I.); honjomegumi@gmail.com (M.H.); fujishiro.tky@gmail.com (T.F.); tetsuya3952@gmail.com (T.T.); taono-tky@umin.ac.jp (T.O.); 2 Miyata eye hospital, Miyazaki 885-0051, Japan; yosai730@gmail.com (Y.M.); miyata@miyata-med.ne.jp (K.M.); 3 Gunma University Initiative for Advanced Research (GIAR), Gunma 371-8511, Japan; obi@gunma-u.ac.jp

**Keywords:** pterygium, sphingosine 1 phosphate, RhoA, human conjunctiva, ultraviolet

## Abstract

Sphingosine 1 phosphate (S1P) is a bioactive lipid that regulates cellular activity, including proliferation, cytoskeletal organization, migration, and fibrosis. In this study, the potential relevance of S1P–Rho signaling in pterygium formation and the effects of ultraviolet (UV) irradiation on activation of the S1P/S1P receptor axis and fibrotic responses were investigated in vitro. Expressions of the S1P2, S1P4, and S1P5 receptors were significantly higher in pterygium tissue than in normal conjunctiva, and the concentration of S1P was significantly elevated in the lysate of normal conjunctival fibroblast cell (NCFC) irradiated with UV (UV-NCFCs). RhoA activity was significantly upregulated in pterygium fibroblast cells (PFCs) and UV-NCFCs, and myosin phosphatase–Rho interacting protein (MRIP) was upregulated, and myosin phosphatase target subunit 1 (MYPT1) was downregulated in PFCs. Fibrogenic changes were significantly upregulated in both PFCs and UV-NCFCs compared to NCFCs. We found that the activation of the S1P receptor–Rho cascade was observed in pterygium tissue. Additionally, in vitro examination showed S1P–rho activation and fibrogenic changes in PFCs and UV-NCFCs. S1P elevation and the resulting upregulation of the downstream Rho signaling pathway may be important in pterygium formation; this pathway offers a potential therapeutic target for suppressing pterygium generation.

## 1. Introduction

Pterygium is defined as a condition with the invasion of wing-shaped chronic proliferative fibrovascular tissue into normal corneal epithelium. Although the precise mechanisms of pterygium formation are still incompletely understood, several pathological conditions, including genetic conditions, anti-apoptotic mechanisms, cytokines and growth factors, extracellular matrix remodeling via matrix metalloproteinases (MMPs), immunological mechanisms, and environmental conditions, have been implicated in the pathogenesis of pterygium [1]. Several previous epidemiological studies have highlighted ultraviolet (UV) radiation as an important initiating factor inducing pterygium onset, as the occurrence rate of pterygium appears to be closely aligned with atmospheric UV intensity [2,3,4,5,6,7,8]. UV radiation is an environmental causative factor [2,3,4,5,6,7,8]; chronic UV irradiation causes pathophysiological changes in limbal basal stem cells and conjunctival fibroblasts [2,9,10]. Along with the change in the characteristics of limbal stem cells and fibroblasts, kinases, several pro-inflammatory cytokines, and growth factors are upregulated in pterygium tissue. These include interleukin (IL)-1, -6, and -8; basic fibroblast growth factor (b-FGF); vascular endothelial cell growth factor (VEGF); and transforming growth factor-β (TGF-β) [11,12,13], which can lead to fibrosis or angiogenesis. Several studies have suggested that UV radiation induces the production of these cytokines in vitro and ex vivo [2,11,14,15].

Aberrant extracellular matrix (ECM) remodeling is a major feature of pterygium, as evidenced by the upregulation of ECM genes, including fibronectin and collagen [1,16,17]. There are many reports of MMP upregulation and the suppressive effects of its inhibitors in pterygium, suggesting the involvement of MMPs in the pathogenesis of pterygium in the form of MMP proteolytic activity [18,19,20,21,22,23,24].

Sphingosine 1 phosphate (S1P) is a bioactive lipid that regulates cellular activity, including proliferation, cytoskeletal organization, migration, and fibrosis [25,26,27,28,29,30]. S1P is synthesized via the phosphorylation of sphingosine by sphingosine kinase (SphK, SphK1, and SphK2) [26,29,30]. Additionally, the expression of SphK is known to be upregulated with inflammatory factors [26,30]. 

These biological changes are related to MMP activation via S1P regulation [31,32,33,34]; additionally, the attenuation of MMP activity through S1P is regulated by the activation of one of the S1P receptors, S1P2 [35,36]. The activation of S1P-S1P2 signaling leads to RhoA phosphorylation; notably, RhoA is from the Rho superfamily of small GTPases (including Rho and Rac).

Transdifferentiation of fibroblasts into myofibroblasts is found in pterygium tissue [37,38], which is associated with the expression of smooth muscle actin (α-SMA) [39]. Enhancement of α-SMA expression leads to the presence of activated fibroblasts with increased synthesis of extracellular matrix (ECM) proteins, growth factors, and integrins [40,41]. RhoA is involved in the transdifferentiation of fibroblasts into myofibroblasts; therefore, the S1P–RhoA pathway may be involved in the pathogenesis of pterygium. However, to date, the relationship between the genesis of pterygium and S1P–RhoA has not been investigated.

In this study, we investigated the involvement of the S1P–RhoA pathway in pterygium tissue, compared to normal conjunctiva, and whether UV irradiation modulates the expression of this pathway and fibrotic properties in cultured pterygium fibroblast cells and normal conjunctival cells. Our results establish a correlation between UV light exposure and S1P–RhoA pathway involvement, providing insight into the pathogenesis of pterygium.

## 2. Results

### 2.1. mRNA Expression of S1PR in Pterygium and Normal Conjunctiva Tissue

**Figure 1:**
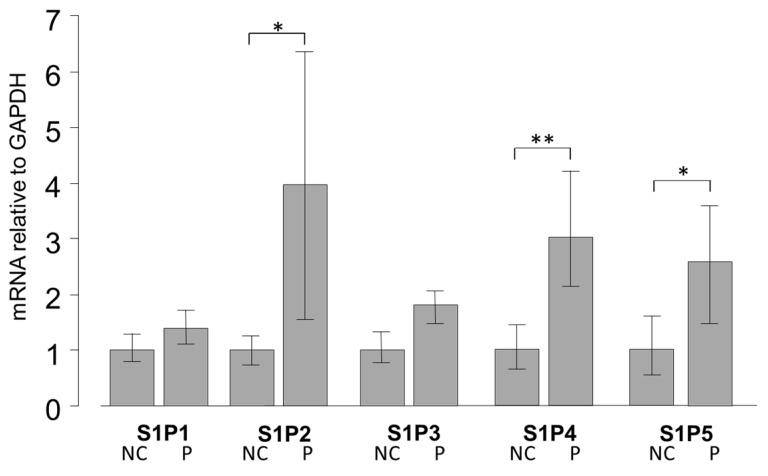
qPCR quantification of normal conjunctiva (NC) tissue and pterygium (P) tissue mRNA expression of S1P1–5 relative to GAPDH (Glyceraldehyde-3-phosphate dehydrogenase) (*n* = 5). The relative mRNA expression of S1P2, 4, and 5 was significantly higher in pterygium tissue than in normal conjunctiva tissue. * *p* < 0.05 and ** *p* < 0.01.

We examined the mRNA expression levels of S1P1–5 in pterygium tissue and normal conjunctival tissue by qRT-PCR (Figure 1). Basal levels of S1P 1–5 were detectable both in pterygium and normal conjunctiva tissues (Figure 1). The relative expressions of mRNA of S1P2 (*p* < 0.05), S1P4 (*p* < 0.01), and S1P5 (*p* < 0.05) were significantly higher in pterygium tissues than in normal conjunctiva tissue.

### 2.2. Dose Effect of UV Light on S1P Production in NCFCs (Normal Conjunctiva Fibroblast Cell)

**Figure 2:**
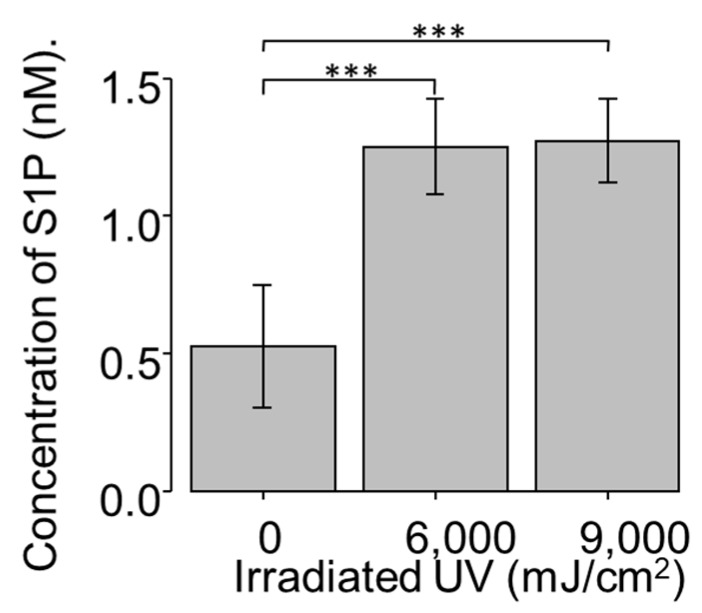
S1P (Sphingosine 1 phosphate) concentration of UV-NCFC (normal conjunctival fibroblast cells irradiated with ultraviolet) lysate (*n* = 4). S1P concentration of cell lysate was significantly upregulated after UV irradiation at 6000 and 9000 mJ/cm^2^. *** *p* < 0.001.

As S1P regulates ECM production, particularly that of collagen and fibronectin, and pterygium features an altered ECM, next, we investigated the relevance between significant expression of S1PR and possible induction of S1P in pterygium tissue. To determine whether S1P production increases with UV treatment, monolayers of confluent NCFCs were irradiated with two doses of UV irradiation: 6000 and 9000 mJ/cm^2^; the concentration of S1P in each group was measured as previously reported [42]. The exposure of NCFCs to UV light resulted in a significant induction of S1P, relative to control values (Figure 2). UV irradiation doses ranging from 0 to 9000 mJ/cm^2^ did not cause any loss in cell viability, as assessed by Trypan Blue exclusion.

### 2.3. Expression of SphK 1 and 2 in NCFCs, PFCs, and UV-NCFCs

**Figure 3:**
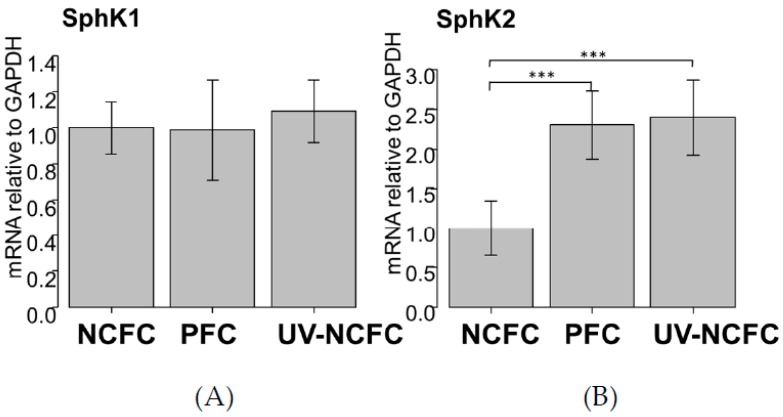
qPCR quantification of NCFC (normal conjunctival fibroblast cell), PFC (pterygium fibrovlast cell), and UV-NCFC (normal conjunctival fibroblast cells irradiated with ultraviolet) mRNA expression of SphK1 (**A**) and SphK2 (**B**) relative to GAPDH (*n* = 4). The relative mRNA expression of SphK2 (**B**) was significantly higher in PFCs and UV-NCFCs than in normal conjunctiva tissue. *** *p* < 0.001.

**Figure 4:**
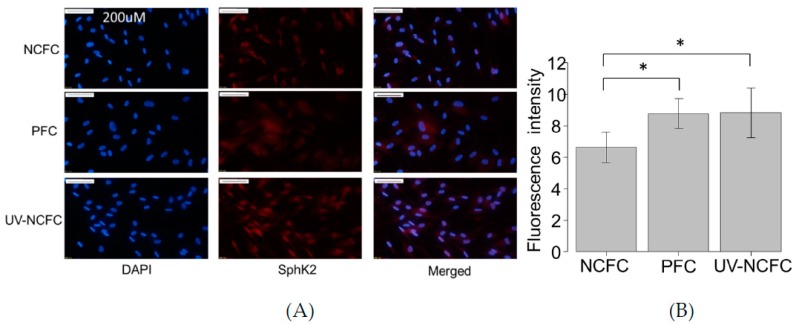
(**A**) Immunocytochemistry of SphK2 in NCFCs, PFCs, and UV-NCFC. The left panels show cells that were stained with DAPI (4’,6-diamidino-2-phenylindole). The middle panels show cells stained for SphK2. The right panels show a merged image. SphK2 was upregulated in PFCs and UV-NCFCs compared to NCFCs. Bar, 200 µm. (**B**) Quantitative results based on immunocytochemistry. Five images of each experiments were taken and the fluorescence intensities were quantified. Data are presented as the mean ± standard deviation. * *p* < 0.05.

As UV irradiation induces significant S1P production and the expression of S1P2 was particularly significant in pterygium tissue, we analyzed the mRNA expression levels of SphK 1 and 2 by qRT-PCR (Figure 3A,B) to determine whether SphK is expressed in UV-NCFCs compared to NCFCs. Relative mRNA expression of SphK2 (*p* < 0.05) was significantly higher in PFCs and UV-NCFCs than in NCFCs. In addition, immunohistochemistry results showed that SphK2 expression was clearly stronger in PFCs and UV-NCFCs than in NCFCs (Figure 4A), and also quantified intensities showed that SphK2 was upregulated in UV-NCFC or PFCs compared to NCFCs (Figure 4B).

### 2.4. Expression of S1P and S1P2R (EDG-5) in NCFCs, PFCs, and UV-NCFCs

**Figure 5:**
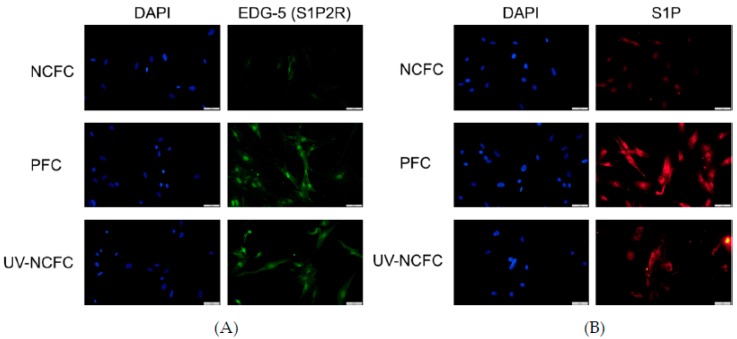
(**A**) Immunocytochemistry of EDG-5 (endothelial differentiation, sphingolipid G-protein-coupled receptor-5) in NCFCs, PFCs, and UV-NCFC. The left panels show cells that were stained with DAPI. The right panels show cells stained for EDG-5. EDG-5 was upregulated in PFCs and UV-NCFCs compared to NCFCs. Bar, 200 µm. (**B**) Immunocytochemistry of S1P in NCFCs, PFCs, and UV-NCFC. The left panels show cells that were stained with DAPI. The right panels show cells stained for S1P. S1P was upregulated in PFCs and UV-NCFCs compared to NCFCs. Bar, 200 µm.

As SphK2 expression was upregulated in PFCs and UV-NCFCs compared to NCFCs, we next performed immunocytochemistry against S1P and S1P2R (EDG-5) in NCFCs, PFCs, and UV-NCFCs. The expression of S1P2R (Figure 5A) and S1P (Figure 5B) was upregulated in PFCs and UV-NCFCs compared to NCFCs.

### 2.5. Effects of UV Irradiation on Rho Activation in NCFCs

**Figure 6:**
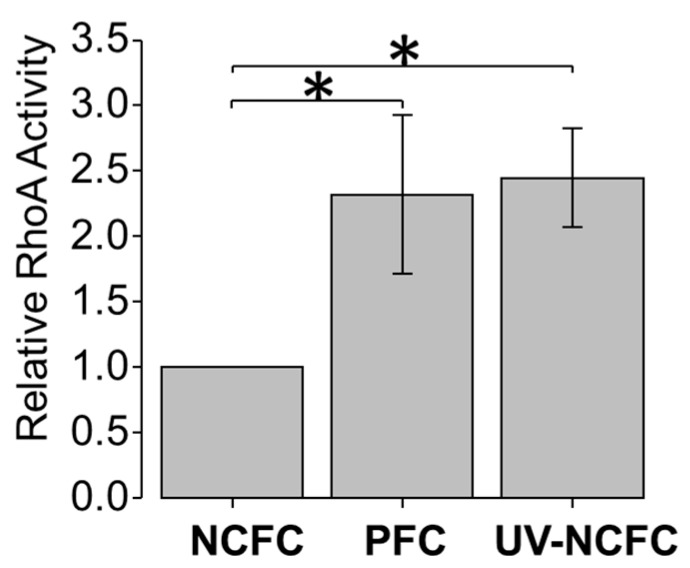
Rho A activation assay for NCFCs, PFCs, and UV-NCFCs (*n* = 3). The amount of activated Rho of NCFCs, PFCs, and UV-NCFCs was determined by a pull-down assay. Data shown in the lower panel represent the relative change in GTP–Rho. Relative RhoA activity increased significantly in PFCs and UV-NCFCs. Data are shown as mean ± SE, * *p* < 0.05 and ** *p* < 0.01.

UV irradiation induced significant S1P production; S1P2 expression was particularly significant in pterygium tissue, which regulates the fibrotic response through Rho signaling as a downstream pathway. As such, we examined the activation of RhoA-ROCK signaling in pterygium tissue and after UV irradiation in NCFCs. A pull-down assay was used to evaluate the strength of active RhoA (GTP–RhoA). There was a marked increase in relative RhoA activity of approximately 2.5-fold (*p* < 0.001) relative to the non-treated control NCFCs after UV irradiation (Figure 6). In addition, RhoA was significantly upregulated in PTFCs (*p* < 0.01) compared to control NCFCs (Figure 6).

### 2.6. Expression of MRIP, MYPT1 in PFCs and NCFCs

**Figure 7:**
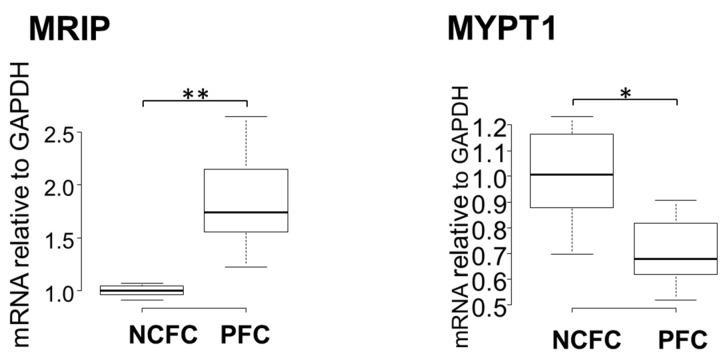
qPCR quantification of NCFCs and PFC mRNA expressions of MRIP and MYPT1 relative to GAPDH (*n* = 4). The relative mRNA expression of MRIP was significantly higher in PFCs than in normal conjunctiva tissue. The relative mRNA expression of MYPT1 was significantly lower in PFCs than in normal conjunctiva tissue. * *p* < 0.05 and ** *p* < 0.01.

A RhoA pull-down assay revealed that the RhoA was significantly activated in pterygium tissue and in NCFCs after UV irradiation. Next, we determined the mRNA expression of the myosin-binding subunit of MYPT1 and MRIP in both PFCs and NCFCs by qRT-PCR. MYPT1 and MRIP are relatively novel members of the myosin phosphatase regulatory complex that directly bind RhoA and regulate myosin light chain (MLC) phosphatase (MLCP), resulting in actin stress fiber assembly [43,44]. As shown in Figure 7, the relative mRNA expression of MRIP was significantly high, whereas the expression of MYPT1 was downregulated in pterygium cells. It has been suggested that MLCP requires enhanced expression and an association with both MRIP and MYPT1 for efficient dephosphorylation of MLC, and MRIP upregulation increases actin stress fibers and MLC phosphorylation [43]. Therefore, the present results indicate that the S1P/S1P2/RhoA pathway is upregulated in pterygium.

### 2.7. Comparing Fibrogenic Changes and Actin Fibers Between Normal Conjunctiva Fibroblasts and Pterygium Conjunctiva Fibroblasts

**Figure 8:**
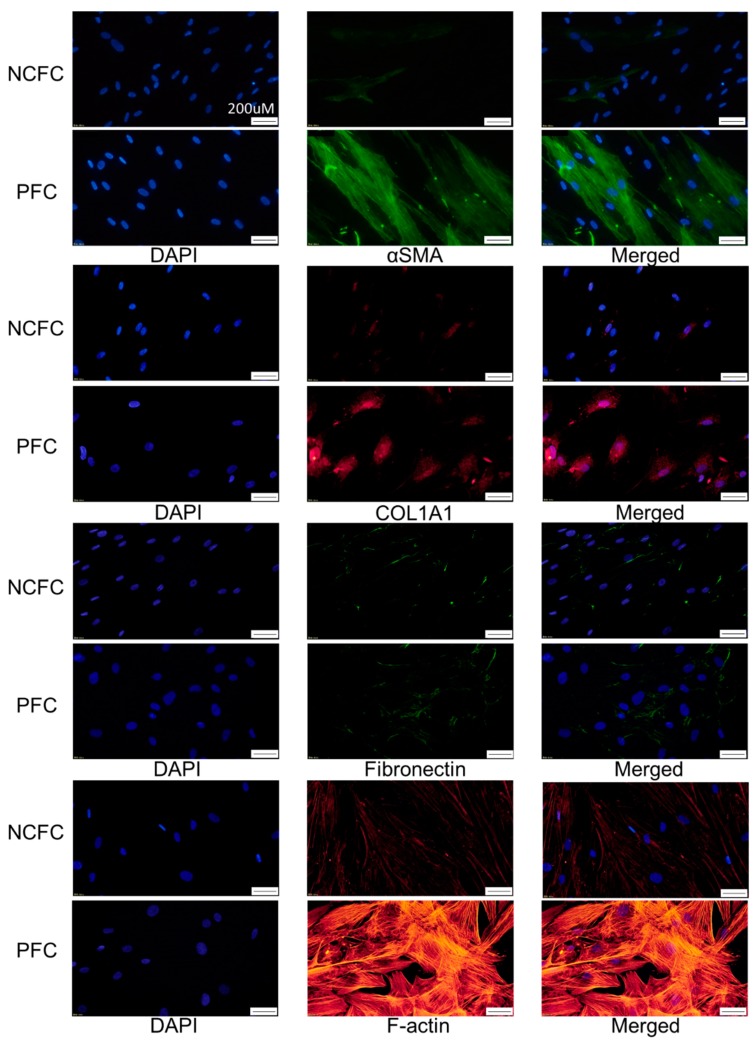
Immunocytochemistry of α-SMA (alpha-smooth muscle actin), COL1A1 (Collagen Type I Alpha 1 Chain), fibronectin, and F-actin in NCFCs and PFCs. The left panels show cells that were stained with DAPI. The middle panels show cells stained for either α-SMA, COL1A1, fibronectin, or F-actin. The right panels show a merged image. These components were upregulated in PFCs compared to NCFCs. Bar, 200 µm.

Immunocytochemistry was used to assess the differences in fibrogenic changes and actin fibers between NCFCs and PFCs. Fibronectin and COL1A1 (Collagen Type I Alpha 1 Chain) expression and phalloidin staining and localization to actin fibers were observed (Figure 8); these effects were stronger in PFCs than in NCFCs.

### 2.8. Effects of UV Irradiation on the Expression of MMP-3 and IL-8 in NCFCs with or without SKI-I

**Figure 9:**
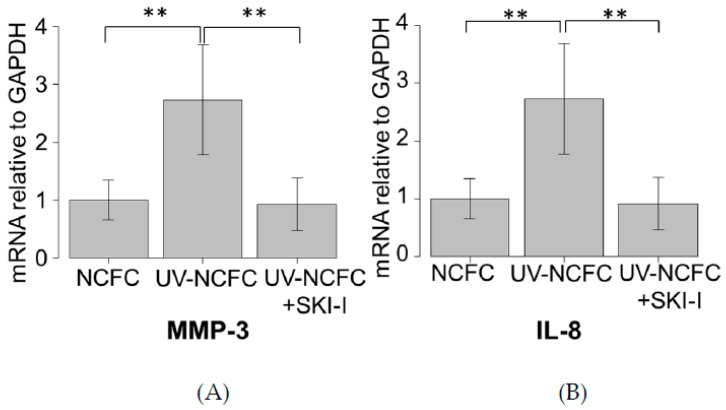
qPCR quantification of NCFCs UV-NCFCs with or without SKI-I. mRNA expressions of MMP-3 and IL-8 relative to GAPDH (*n* = 4). The relative mRNA expression of MMP-3 and IL-8 was significantly higher in UV-NCFCs than in NCFCs. ** *p* < 0.01.

We further investigated if UV irradiation induces the expression of downstream cascade of S1P. We analyzed the mRNA expression levels of MMP-3 and IL-8 by qRT-PCR (Figure 9A,B) to determine whether MMP-3 and IL-8 is expressed in UV-NCFCs compared to NCFCs. Additionally, we also assessed whether SKI-I can attenuate the expression of MMP-3 and IL-8 when NCFCs were irradiated with UV. Relative mRNA expression of MMP-3 and IL-8 (*p* < 0.01) was significantly higher in UV-NCFCs than in NCFCs.

### 2.9. Effects of UV Irradiation on Cytoskeletal Changes and Fibrotic Responses in NCFCs

**Figure 10:**
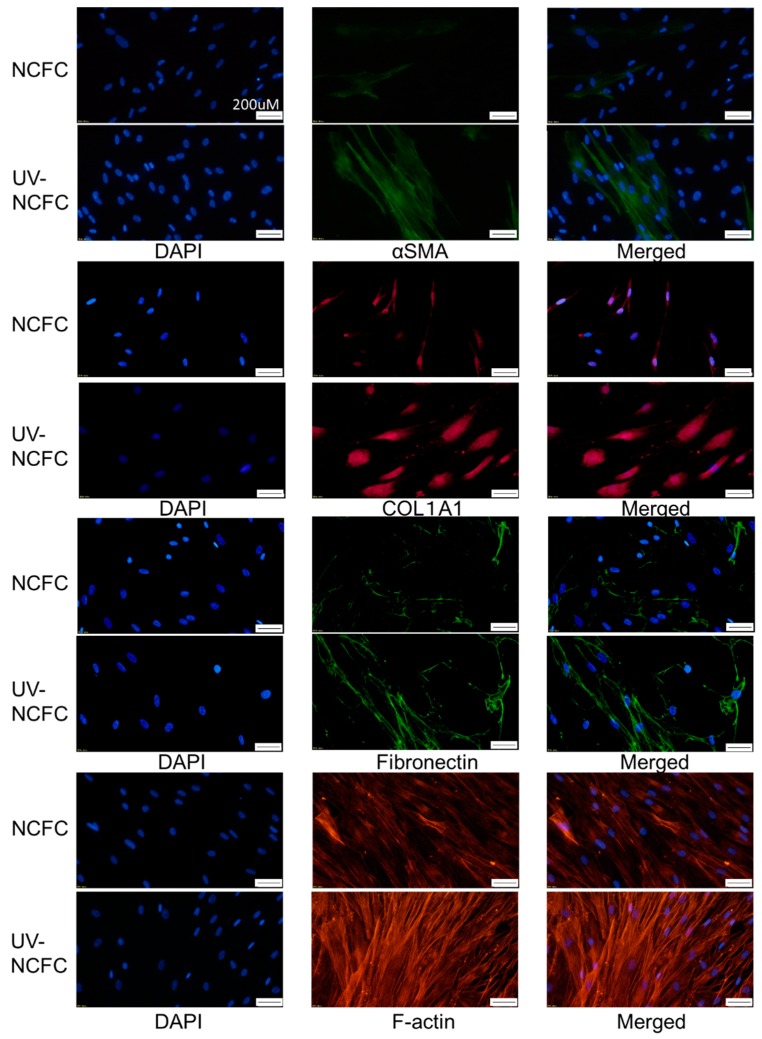
Immunocytochemistry of α-SMA, COL1A1, fibronectin, and F-actin in NCFCs and UV-NCFCs. The left panels show cells that stained with DAPI. The middle panels show cells stained for either α-SMA, COL1A1, fibronectin, or F-actin. The right panels show a merged image. These components were upregulated in UV-NCFCs compared to NCFCs. Bar, 200 µm.

**Figure 11:**
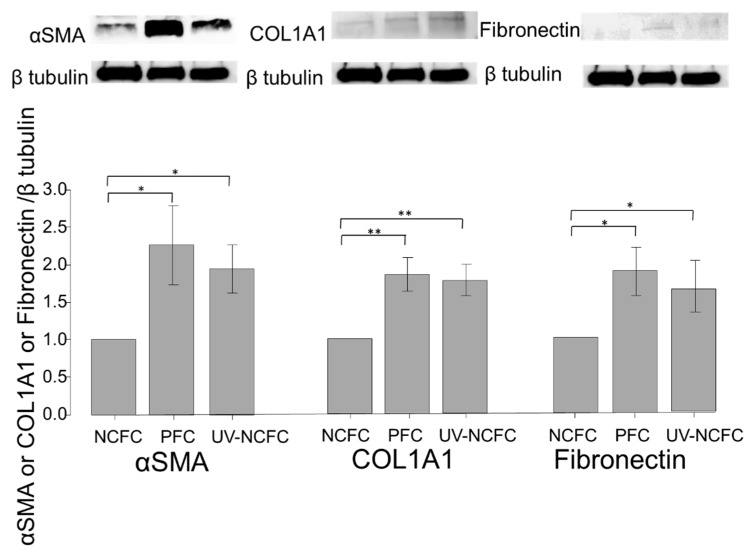
Western blotting of fibronectin, COL1A1, and α-SMA in NCFCs, PFCs, and UV-NCFCs (*n* = 3). Relative expression of α-SMA, COL1A1, and fibronectin. Results are expressed relative to the loading control (β-tubulin). These components were upregulated in PFCs and UV-NCFCs compared to NCFC. * *p* < 0.05, ** *p* < 0.01.

Finally, we investigated whether UV irradiation induces cytoskeletal changes or a fibrotic response in NCFCs. Immunocytochemistry and western blotting were used to assess changes induced by UV irradiation. The expressions of fibronectin, COL1A1, and phalloidin and α-SMA staining were significantly upregulated in NCFCs after UV irradiation (Figure 10). Also, Figure 11 shows that the expression of fibronectin, COL1A1, and α-SMA was significantly upregulated in NCFC after UV irradiation with western blotting (WB) assessment.

### 2.10. Comparing Fibrogenic Changes and Actin Fibers Between Normal Conjunctiva Fibroblasts and Ultraviolet Irradiated Normal Conjunctiva Fibroblast with or without JTE-013

**Figure 12:**
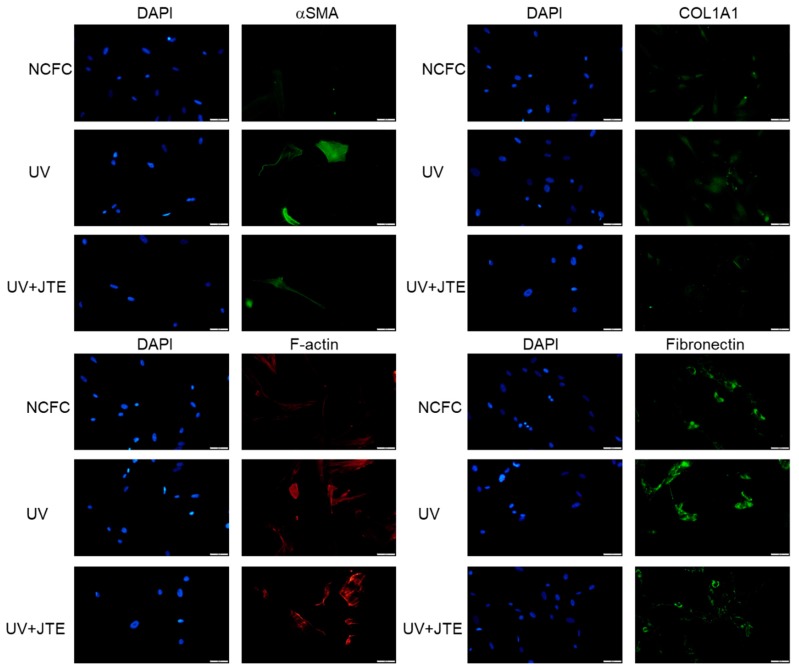
Immunocytochemistry of α-SMA, COL1A1, fibronectin, and F-actin in NCFCs and UV-NCFCs with or without JTE-013. The left panels show cells stained with DAPI. The right panels show cells stained for either α-SMA, COL1A1, fibronectin, or F-actin. These components were upregulated in UV-NCFCs compared to NCFCs, and those changes were attenuated with JTE-013. Bar, 200 µm.

We finally examined if the fibrogenic responses induced with UV irradiation would be attenuated with JTE-013. As shown in Figure 12, the expression of aSMA, COL1A1, F-actin, and fibronectin was upregulated with UV irradiation, and downregulated with JTE-013.

## 3. Discussion

S1P is a bioactive lipid that regulates cellular activities, such as proliferation, cytoskeletal organization, migration, and fibrosis, by attenuating downstream cascades, including cytokine and MMP activities [25,26,27,28,29,30]. The activity of S1P is controlled by one of the S1P receptors, S1P2, and the RhoA-ROCK pathway, which is the downstream cascade of S1P-S1P2 signaling [35,36]. The RhoA–ROCK pathway also regulates actomyosin cytoskeletal organization, cell adhesion, ECM production, and cell motility [45,46,47,48]. Many studies have shown a strong association between the S1P–rhoA pathway and tumor progression/invasion [49]; thus, the invasive nature of the pterygium lesion is likely to involve altered RhoA activity. However, the roles of S1P and the RhoA cascade in the genesis of pterygium have not been evaluated in human pterygium (until now).

In our study, we first detected the expression of S1P receptors in both normal conjunctiva tissue and pterygium tissue. The relative mRNA expressions of S1P2 (*p* < 0.05), S1P4 (*p* < 0.01), and S1P5 (*p* < 0.05) were significantly higher in pterygium tissues than in normal conjunctiva tissue (Figure 1), suggesting the involvement of S1P activation, along with the S1P receptors mentioned above, in the genesis of pterygium.

Epidemiological studies have shown that chronic UV irradiation contributes to the growth and development of pterygium; notably, many kinds of pro-inflammatory cytokines are involved in the generation of pterygium, and they are induced by UV irradiation [2]. Therefore, we explored whether UV irradiation induces S1P production in normal conjunctiva fibroblasts. Exposure of NCFCs to UV irradiation resulted in a significant induction of S1P, relative to control values (Figure 2). The S1P cascade has many downstream signaling channels, including RhoA, which is activated via the S1P2 receptor [36]. Thus, from the present data, we postulate that UV irradiation causes S1P production in conjunctiva tissue, resulting in the upregulation of RhoA to generate pro-inflammatory cytokines and kinases, such as MMPs. Such cytokines and MMPs may promote changes in the characteristics of conjunctival tissue or limbal tissue, resulting in pterygium development.

We observed upregulation of S1P receptors and S1P concentration after UV irradiation. As such, next, we assessed SphK expression in NCFCs, PFCs, and UV-NCFCs. The SphK enzyme catalyzes the ATP-dependent phosphorylation of sphingosine into S1P, and the two isozymes SphK1 and SphK2 have been identified in mammals [50]. As shown in Figure 3A,B, relative mRNA expression of SphK2 was significantly higher in PFCs and UV-NCFCs than in NCFCs (*p* < 0.05). Immunocytochemistry also supported these data (Figure 4A,B).

As SphK2 expression was upregulated in PFCs and UV-NCFCs, we further compared the expression of S1P and S1P2R in NCFCs, PFCs, and UV-NCFCs. Immunocytochemistry against S1P and S1P2R (EDG-5) in NCFCs, PFCs, and UV-NCFCs showed that the expression of S1P and S1P2R was upregulated in PFCs and UV-NCFCs compared to NCFCs (Figure 5A,B).

To confirm the relationship between S1P upregulation and activation of the downstream RhoA cascade, we performed a RhoA pull-down assay; we observed a marked increase in the relative RhoA activity of approximately 2.5-fold after UV irradiation (*p* < 0.001) with respect to nontreated control NCFCs (Figure 6). RhoA was significantly upregulated in PTFCs (*p* < 0.01) compared to control NCFCs (Figure 6). From our present results, we can assume that UV induces S1P upregulation and activates downstream RhoA signaling, possibly through the S1P2 receptor. The accumulated amount of irradiated UV in conjunctiva may result in continuous expression of the RhoA cascade, causing the conjunctiva tissue to form pterygium via the sustained high levels of pro-inflammatory cytokines.

To support the hypothesis regarding the involvement of RhoA signaling in the genesis of pterygium, next, we explored MRIP, a protein that interacts directly with RhoA and its substrate (MYPT1, an endogenous inhibitor of MLCP) [51,52,53,54,55,56]. Generally, Rho kinase inactivates MLCP by phosphorylating MYPT1 [57]. In previous studies, MRIP silencing led to the inhibition of LPA (lysophosphatidic acid)-mediated phosphorylation of MYPT1, resulting in a contractile response in cells [58]. Thus, MRIP is assumed to control RhoA activity and MLCP [59]. In our study, mRNA expression of MRIP was upregulated, whereas MYPT1 was downregulated in fibroblasts derived from pterygium, compared to fibroblasts derived from normal conjunctiva (Figure 7). This suggests that upregulated MRIP accelerates phosphorylation of MYPT1 in fibroblasts derived from pterygium. Further studies are needed to determine the involvement and reaction of precise pathways downstream of RhoA.

We also assessed and compared the fibrogenic changes in NCFCs, PFCs, and UV-NCFCs. Figure 8 shows the differences between NCFCs and PFCs, and Figure 10 shows the differences between NCFCs and UV-NCFCs based on immunocytochemistry. Fibronectin and COL1A1 expression and phalloidin staining and localization to actin fibers were also clearly observed and were stronger in PFCs and UV-NCFCs than in NCFCs. Western blotting (Figure 11) also showed the same trend, with significant expressions of fibronectin, COL1A1, and α-SMA in PFCs and UV-NCFCs compared to NCFCs. Taken together with the upregulation of RhoA in PFCs and UV-NCFCs, these results indicate upregulated fibrogenic changes in UV-irradiated fibroblasts, as well as in fibroblasts derived from pterygium; this also suggests common pathological conditions in these fibroblasts, including α-SMA enhancement and increased synthesis of ECM proteins [40,41].

We also performed experiments to ascertain whether the UV induced effect can be downregulated with SphK inhibitor or S1P2R inhibitors. Figure 9 shows the results of qPCR quantification of NCFCs UV-NCFCs with or without SKI-I, and the figure shows that the expression of MMP-3 or IL-8, which are known pro-inflammatory substances produced by the activation of RhoA, were significantly upregulated with UV irradiation, and those changes were attenuated with SKI-I. Also, the inhibition of S1P2R resulted in the attenuation of UV-induced fibrogenic changes (Figure 12).

Our study had several limitations. First, the ages of the patients providing pterygium and normal conjunctiva tissues were not uniform; thus, upregulated RhoA in pterygium tissue may not only be due to accumulated UV irradiation but also may be affected by aging or other genetic confounding factors. Second, we were unable to completely resolve the precise mechanism of SphK1 induction or contributions to proinflammatory cytokine expression in conjunctiva after UV irradiation. As previously reported, UV irradiation itself may result in the upregulation of several cytokines. SphK has pleiotrophic effects and may regulate cytokine expression [60,61]; thus, further study is needed to determine the underlying mechanisms and crosstalk. Third, the usual location of pterygium is at the nasal part of the cornea; however, we did not examine the correlation between S1P and where the formation part of pterygium is. Coroneo et al. reported the coincidence of pterygium location with an intense nasal light focus, which would support our hypothesis. Higher UV intensity in the nasal area may lead to the accumulation of S1P induced by UV irradiation, causing dysfunction of limbal stem cells and conjunctival damage to form pterygium. Fourthly, we did not perform in vivo experiments using animals to assess whether the inhibition of S1P could be a novel treatment for pterygium, so further studies would be needed to confirm the effect of S1P on the formation of pterygium. Finally, we did not fully explore the downstream pathway of RhoA. As ROCK inhibition could suppress MMP-3 production in chondrocytes [62], and we found that UV-induced expression of MMP-3 and IL-8 were attenuated with SKI-I in qPCR, we will further investigate the involvement of ROCK and MMPs in future research. 

**Figure 13:**
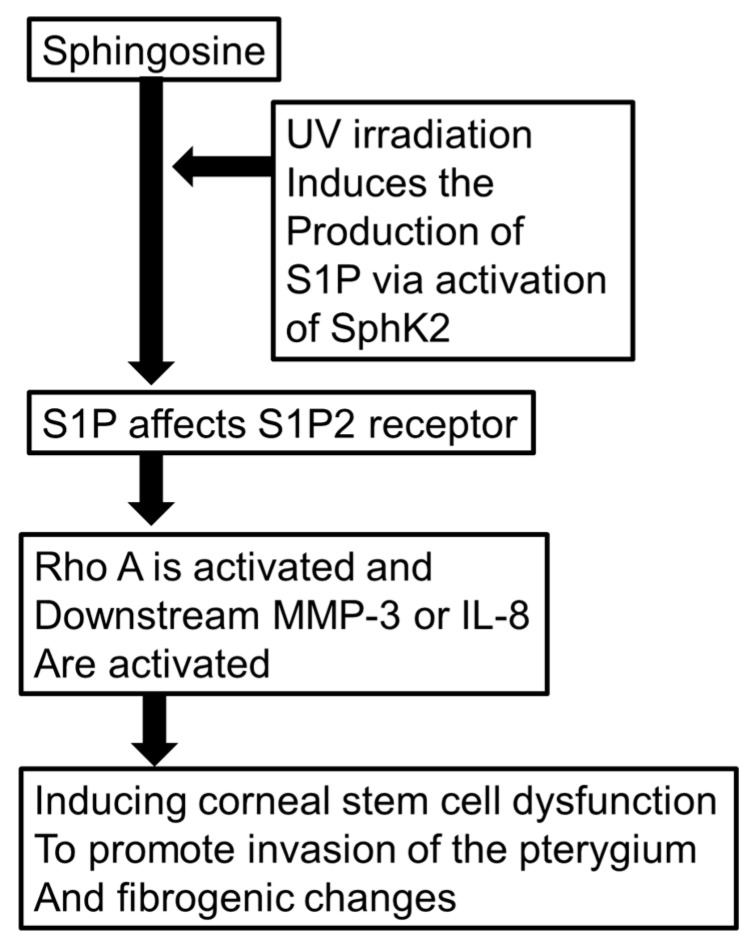
Schematic presentation of signaling cascade/hypothetic SphK2/S1P2 receptor pathway in the formation of pterygium.

In conclusion, we hypothesized that the RhoA cascade is upregulated in pterygium tissue and may play an important role in the genesis of pterygium. As shown in Figure 13, we hypothesized that UV irradiation induces the production of S1P via activation of SphK2 to produce S1P. Additionally, S1P upregulation induced by UV irradiation was assumed to be a factor promoting RhoA upregulation and its downstream MMP-3 or IL-8 to facilitate the onset of pterygium. Therefore, the inhibition of this cascade may be a useful alternative approach to clinical management of pterygium. Further studies are necessary to understand the precise molecular mechanisms.

## 4. Materials and Methods

### 4.1. Patients and Samples from Patients Who Underwent Surgery for Pterygium, Strabismus, or Cataract

Pterygium tissues were obtained from patients who underwent pterygium excision surgery at the University of Tokyo Hospital and Miyata Eye Hospital. The protocol of this study was approved by the institutional review board of University of Tokyo (YWMR-15-0-053, 26 October 2015). All subjects were treated, and all procedures were performed, in accordance with the Declaration of Helsinki. Written informed consent to participate was obtained from each patient. Exclusion criteria included patients with recurrent pterygium. The head part of the pterygium was used in all experiments, as the invading character differs between the head and body part of the pterygium [63]. Normal conjunctiva samples were collected from patients who underwent strabismus operation or cataract surgery. Normal conjunctiva and pterygium tissues were collected from different patients.

### 4.2. Isolation and Culture of Primary Pterygium and Normal Conjunctiva Fibroblast Cells from Patients

Resected pterygium or normal conjunctiva tissue were embedded in Cellmatrix Type I-A (Nitta Gelatin Inc., Osaka, Japan) mixed with Ham’s F-12 medium and reconstitution buffer (both Nitta Gelatin Inc.), at an 8:1:1 ratio, and placed in a 35-mm cell culture dish. Dulbecco’s modified Eagle’s medium (DMEM) containing 10% fetal bovine serum (FBS) and antibiotic antimycotic solution (100×) (Sigma-Aldrich 136 Co., LLC, St. Louis, MO, USA) were added to cover the tissue. The culture dish was placed in a CO_2_-regulated incubator with a 5% CO_2_ atmosphere; 2 mL medium was changed every 2 days thereafter. After an outgrowth of cells from the tissue was observed, collagenase L was added to dissolve the collagen medium. Then, the test specimens were incubated in a 5% CO_2_ atmosphere incubator for 30 min, which was followed by treatment with 0.1% trypsin and 0.02% ethylenediaminetetraacetic acid (EDTA). The dissolved medium was collected and centrifuged to collect cells. Isolated cells from passages 3–6 were used in the experiments.

### 4.3. UV Irradiation

Normal conjunctiva fibroblast cells (NCFCs) or pterygium fibroblast cells (PFCs) were grown to confluence in 10% FBS DMEM. After 24 h of starvation of the prepared cells, the medium was replaced with PBS (-) to prepare UV-irradiated normal conjunctiva fibroblast cells (UV-NCFCs). PBS (-) was replaced with FBS (-) DMEM soon after UV irradiation. UV-NCFCs were irradiated with 365 nm 1000 μW/cm^2^ UV irradiation, using a handheld UV lamp (4-W UVL-21 UVP Compact UV Lamps, Funakoshi, Tokyo, Japan). UVA irradiance was measured with a UV meter (UV-340C; Custom, Tokyo, Japan). Cells were irradiated for 30 minutes to obtain a UVA dosage of 6000 mJ/cm^2^ (equal to 15 h of sun radiation [64]). The cells were only irradiated with 9000 mJ/cm^2^ to assess S1P concentration in NCFCs; otherwise, the value above was used.

### 4.4. RhoA Activation Assay

Five minutes after UV irradiation, RhoA activation was examined via the pull-down assay using the Rho Activation Assay Biochem kit (Cytoskeleton, Denver, CO, USA), according to the manufacturer’s instructions. Horseradish peroxidase (HRP)-conjugated secondary antibody (1:1000) was purchased from Thermo Fisher Scientific (Waltham, MA, USA). Protein bands were detected using an ImageQuant LAS 4000 Mini system (GE Healthcare, Chicago, IL, USA). The bands were quantified using Adobe Photoshop (Adobe Inc., San Jose, CA, USA).

### 4.5. Quantitative Real-Time PCR

For comparison of the expression of S1P receptors between pterygium and normal conjunctiva tissue, both tissues isolated from normal conjunctiva and pterygium were lysed using Isogen (Nippon Gene, Tokyo, Japan). For experiments with cultured cells, NCFCs and PFCs were grown to confluence, starved, and lysed using TRI Reagent (Molecular Research Center, Inc., Cincinnati, OH, USA). mRNA was isolated using chloroform and isopropyl alcohol and then treated with a PrimeScript RT Reagent Kit (Takara Bio, Shiga, Japan) to synthesize cDNA. mRNA levels were quantified using quantitative PCR (qPCR) of cDNA as previously described [65]. The sequences of the PCR primers are shown in Table S1. Data were normalized relative to GAPDH.

### 4.6. Effect of UV Irradiation on Concentration of S1P in NCFC

Normal conjunctiva fibroblasts isolated from patients were exposed to UV irradiation of 0, 6000, or 9000 mJ/cm^2^. Four hours after irradiation, cells were lysed in 200 µL methanol and stirred for 30 min, followed by lysate collection. S1P quantification was performed as previously described [42].

### 4.7. Immunocytochemistry

Eight hours after irradiation, cells were fixed in ice-cold 4% paraformaldehyde for 15 min, permeabilized with 0.3% Triton X-100 for 5 min, and blocked in 3% bovine serum albumin for 30 min. Immunocytochemistry was performed as previously described [65]. The primary antibodies were anti-fibronectin (1:400; Abcam, Cambridge, MA, USA), anti-collagen type I (1:400; Cell Signaling Technology, Danvers, MA, USA), anti-rhodamine phalloidin (7:1000; Thermo Fisher Scientific, Waltham, MA, USA), anti-αSMA (1:500; Sigma-Aldrich Co., LLC St. Louis, MO USA) and anti-SphK (Sphingosine kinase) 2 (1:400; Abcam, Cambridge, MA, USA), anti-EDG5 (S1PR2) (1:200; Santa Cruz Biothechnology, Dallas, TX, USA), and anti-S1P (1:200; Abcam, Cambridge, MA, USA).

Alexa Fluor 488 and 594 secondary antibodies (1:1000) were purchased from Thermo Fisher Scientific. Cells were counter stained with 4′,6-diamidino-2-phenylindole (DAPI). Quantitative results based on immunocytochemistry were quantified with ImageJ 1.49 (NIH Bethesda, MD, USA). Five images of each experiments were taken and the fluorescence intensities were quantified.

### 4.8. Western Blotting

Eight hours after irradiation, cells were collected in RIPA Buffer (Thermo Fisher Scientific, Kanagawa, Japan) containing protease inhibitors (Roche Diagnostics, Basel, Switzerland), sonicated, and then centrifuged. The following procedures were performed as previously described [65]. Primary antibodies were anti-αSMA (Sigma-Aldrich Co., LLC St. Louis, MO, USA, 1:1000), anti-β-tubulin (Wako Pure Chemical Industries, Ltd., Osaka, Japan, 1:1000), anti-fibronectin (1:1000; Abcam, Cambridge, MA, USA), and anti-collagen type I (1:1000; Cell Signaling Technology, Danvers, MA, USA). HRP-conjugated secondary antibody (1:2000) was purchased from Thermo Fisher Scientific (Waltham, MA, USA). The bands were quantified with ImageJ 1.49 (NIH Bethesda, MD, USA).

### 4.9. Statistical Analysis

Data were statistically analyzed using the EZR program (Saitama Medical Center, Hidaka, Japan) [66]. The results are expressed as means ± standard deviations (SDs). The *t*-test and chi-square or Fisher’s exact test were used to compare two variables, and the Steel–Dwass test was used for multiple variables. Differences in data among the groups were analyzed by one-way analysis of variance (ANOVA) and Tukey’s test was used as a post-hoc test. A value of *p* < 0.05 was considered statistically significant.

## Supplementary Materials

Supplementary materials can be found at https://www.mdpi.com/1422-0067/20/19/4670/s1. Table S1 shows the sequences of the PCR primers used in this manuscript.
