# Supplementary material for: Activation of the Sphingosine 1 Phosphate–Rho Pathway in Pterygium and in Ultraviolet-Irradiated Normal Conjunctiva
Source: Int J Mol Sci. 2019 Sep 20;20(19):4670. doi: 10.3390/ijms20194670 (PMC6801701; doi:10.3390/ijms20194670)
Supplement: Supplementary file 1 [file ijms-20-04670-s001.pdf]

Table 1. The sequences of the PCR primers.

PCR Primers	Forward	Reverse
GAPDH	5′-GAGTCAACGGATTTGGTCGT-3′	5′-TTGATTTTGGAGGGATCTCG-3′
S1P1	5′- AAATTCCACCGACCCATGTA -3′	5′- AGTTATTGCTCCCGTTGTGG -3′
S1P2	5′- ACTGTCCTGCCTCTCTACGCC -3′	5′- GTCTTGAGCAGGGCTAGCGTC -3′
S1P3	5′- ACCATCGTGATCCTCTACGCAC -3′	5′- CTTGATTTACTTCTGCTTGGGTCG -3′
S1P4	5′- TGCTGAAGACGGTGCTGATG -3′	5′- CCCAGAGGTTGGAGCCAAAG -3′
S1P5	5′- AGGACCTTGTGGGTGATATAGAGGAC -3′	5′- CCCCTTCACCTTCTCTGGTTTTC -3′
MRIP	5′- CTCTCCACACACGAGCTGAC -3′	5′- TCTTCTGGTGCGTTTCTTCC -3′
MYPT1	5′- AACGACGACGACCAAGAGAG -3′	5′- CCCAGCAAGGAATCATATCG -3′
SphK1	5′- CATTATGCTGGCTATGAGCAG -3′	5′- GTCCACATCAGCAATGAAGC -3′
SphK2	5′- GGTTGCTTCTATTGGTCAATCC -3′	5′- GTTCTGTCGTTCTGTCTGGATG -3′
